# Changes in free amino acid and protein polymerization in wheat caryopsis and endosperm during filling after shading

**DOI:** 10.3389/fpls.2024.1344972

**Published:** 2024-02-15

**Authors:** Hongliang Ma, Yongheng Yang, Dongming Wu, Gang Xiang, Ting Luo, Xiulan Huang, Hongkun Yang, Ting Zheng, Gaoqiong Fan

**Affiliations:** ^1^ Key Laboratory of Crop Eco-Physiology and Farming System in Southwest China, Ministry of Agriculture and Rural Affairs, Chengdu, China; ^2^ Crop Ecophysiology and Cultivation Key Laboratory of Sichuan Province, Sichuan Provincial Department of Agriculture and Rural Affairs, Chengdu, China; ^3^ College of Agronomy, Sichuan Agricultural University, Chengdu, China; ^4^ State Key Laboratory of Crop Gene Exploration and Utilization in Southwest China, Ministry Science and Technology, Chengdu, China

**Keywords:** wheat, endosperm, shade stress, protein polymerization, free amino acid

## Abstract

Over the past several decades, a decreasing trend in solar radiation has been observed during the wheat growing season. The effects of shade stress on grain yield formation have been extensively studied. However, little information on shade stress’s effects on protein formation warrants further investigation. Two wheat cultivars were grown under three treatments, no shade as the control group (CK), shading from the joint to the anthesis stage (S1), and shading from the joint to the mature stage (S2), to investigate the effects of shade stress on the free amino acids of the caryopsis and endosperm and protein accumulation during grain filling. The dry mass of caryopsis and endosperm was significantly decreased under shade stress, whereas Glu, Ser, Ala, and Asp and protein relative content increased during grain filling. The observed increases in total protein in S1 and S2 were attributed to the increases in the SDS-isoluble and SDS-soluble protein extracts, respectively. S1 improved polymer protein formation, but S2 delayed the conversion of albumins and globulins into monomeric and polymeric proteins. Moreover, shade stress increased the proportion of SDS-unextractable polymeric protein, which represented an increase in the degree of protein polymerization. The polymerization of protein interrelations between protein components and accumulation in caryopsis and endosperm provided novel insights into wheat quality formation under shade stress.

## 1 Introduction

Cereal endosperms are important food sources, and their content is a major determinant of yield and end-use quality (Shewry and Morell, 2001; Liu et al., 2022). Wheat is commonly milled to produce white flour. The starchy endosperm of wheat accounts for approximately 80% of the dried weight of the grain, and millers strive for white flour yields that are similar to this value (Tosi et al., 2018).

Wheat grain and flour proteins are essential nutritional traits and significant factors in determining end-use quality (Dowell et al., 2008). The starchy endosperm contains the major gluten storage proteins (gliadins and glutenins), as well as albumin and globulin (Bechtel et al., 2009). During grain development, the supply of free amino acids and the capacity for storage protein synthesis interact to control protein accumulation and content in mature grains (Zheng et al., 2022), and grain protein may be further limited by amino acid supply during grain filling (Zhong et al., 2020). Extractability of proteins in the SDS buffer, which may represent the protein polymerization process, was assessed using SE-HPLC (Hayta and Schofield, 2004). During grain maturation, proteins have been demonstrated to polymerize gradually: from albumins and globulins to large monomers; small SDS-soluble polymers; and, finally, larger and insoluble polymers (Stone and Nicolas, 1996; Ferreira et al., 2012). In wheat grains, monomeric and polymeric proteins are accumulated concurrently in the central endosperm (Tosi et al., 2011). Wheat flour characteristics and uses are largely determined by the distribution of monomeric and polymeric proteins and the solubility of these proteins (Daniel and Triboi, 2002). Therefore, the investigation of the relationship between free amino acid supply and monomeric and polymeric protein aggregation processing during grain filling will provide valuable information.

For human nutrition, end-use functional qualities, and commodity value, wheat grain quality must be maintained despite climatic change (Nuttall et al., 2017; Pramanick et al., 2022). Previous study has shown that 80% of the 430 stations in various wheat regions across China experienced a decreasing trend in solar radiation during growing season from 1980 to 2020 (Han et al., 2023). Notably, solar radiation has had a significant decreasing trend in the wheat growing season in these stations with a monsoon-influenced humid subtropical climate and a subtropical monsoon climate in China (Li and Tao, 2022). Wheat productivity is significantly reduced by the decrease in solar radiation due to cloudy and rainy weather in the Sichuan Basin of China with wet climates (Yang et al., 2013; Qin et al., 2020). Wheat plants are exposed to long-term low radiation in tree-wheat intercropping systems (Qiao et al., 2019; Jia et al., 2021). Moreover, the grain number per spike decreased by shading before anthesis, the grain weight significantly reduced by shading after anthesis, and the two kinds of shading enhanced the grain protein (Artru et al., 2017; Jia et al., 2021; Yang et al., 2023a). Besides, the concentration of the SDS-insoluble polymer protein increased by shading from joint to maturity during grain filling and in maturity (Li et al., 2012). However, further in-depth studies are required to investigate protein formation in the caryopsis and endosperm during filling under shade stress. It will contribute to further understanding of wheat’s quality formation mechanism under shade stress.

Our previous studies indicated that shading stress affects the protein composition in wheat flour, ultimately leading to changes in baking quality (Ma et al., 2024). In this study, we performed field trials using two wheat cultivars under different shading conditions with a polyethylene screen that reduced the light intensity by 50% across the two consecutive wheat growing seasons. The aim of this study was to evaluate t alterations in (1) the accumulation and polymerization of proteins, (2) the levels of free amino acids (FAAs) in caryopsis and endosperm during grain filling after shade stress, and (3) those relationship between them. Our results advance the understanding of the mechanisms involved in wheat quality under shade stress.

## 2 Methods

### 2.1 Material and field experiments

**Figure 1:**
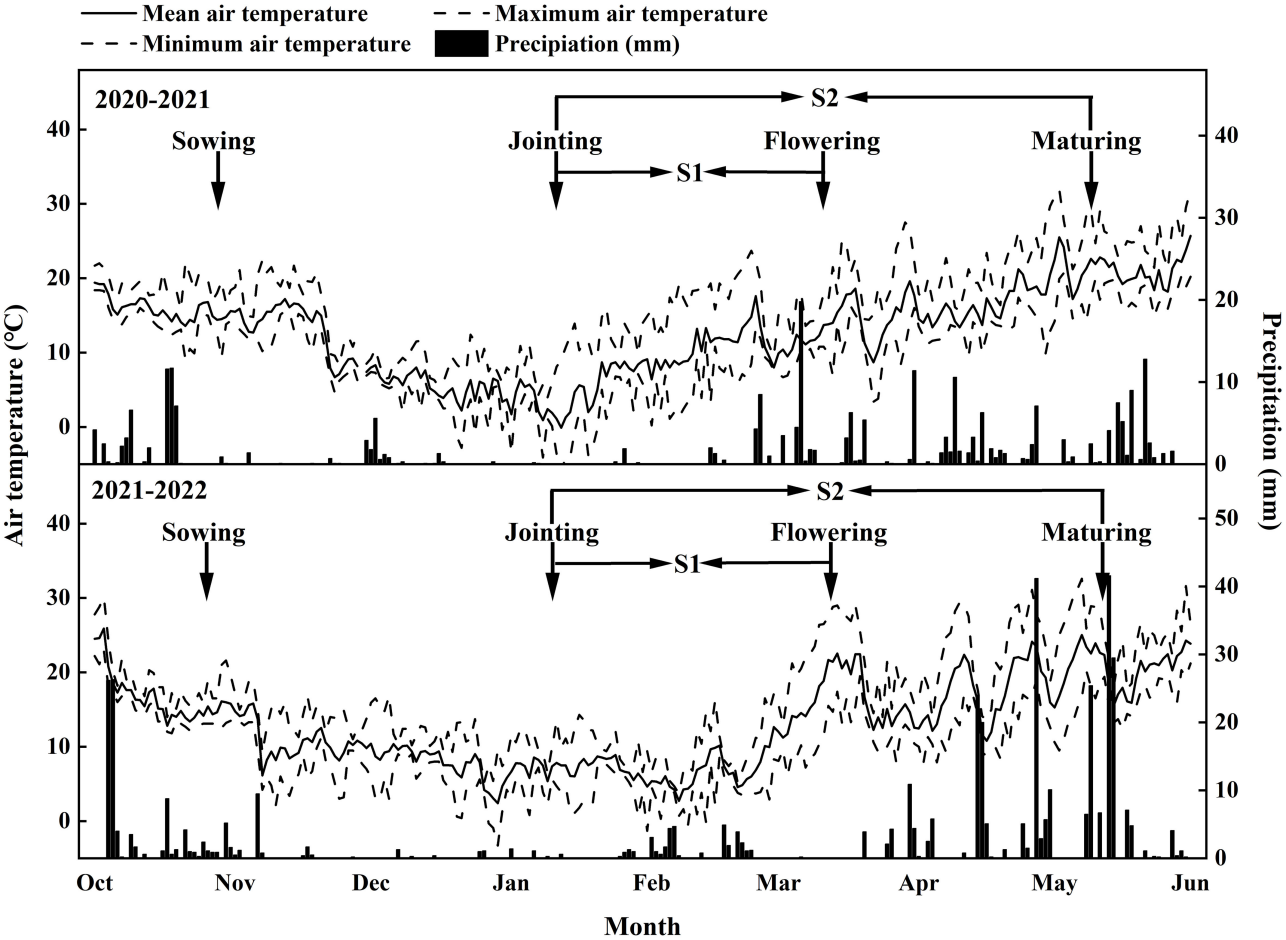
Climate date during the experimental period.

During the 2020.10–2021.5 and 2021.10–2022.5 wheat growing seasons, two locally adapted common wheat (*Triticum aestivum* L.) cultivars, Shumai482 (SM482) and Chuanmai39 (CM39), were grown at the Wenjiang (30°43′N, 103°52′E) experimental stations of Sichuan Agricultural University. The soil was a medium loam with 30.3 g kg^-1^ organic matter, 1.04 g kg^-1^ total N, 0.79 g kg^-1^ total P, 15.1 g kg^-1^ total K, 103.5 mg kg^-1^ alkali hydrolyzable N, 30.4 mg kg^-1^ Olsen-P, and 107.8 mg kg^-1^ exchangeable K. The field experiment was conducted using a split-block design with three replicates. Each plot size was 3 m × 4 m. Seeding density was 250 seeds m^-2^ with 20 cm row spaces. According to local wheat management, 90 kg N ha^−1^, 75 kg P_2_O_5_ ha^−1^, and 75 kg K_2_O ha^−1^ were applied before sowing, and another 60 kg N ha^−1^ was administered at stem elongation. The two cultivars were evaluated under three shading conditions: unshaded (control; CK), partially shaded (S1) from the joint to the anthesis stage, and fully shaded (S2) from the joint to the maturity stage. The polyethylene screen was blocked approximately 50% of the light intensity, which was suspended more than 2 m above the ground to promote excellent ventilation, and completely covered the corresponding shaded plots from the joint to anthesis stage and joint to maturity stage. The timing of the shading was shown in **Figure 1**, as well as the climate data such as daily temperature(mean, maximum and minimum) and rainfall (**Figure 1**).

### 2.2 Sampling

For samples collected when the grains were still developing (14–42 d after anthesis [DAA]), 10–14 grains from the first and second florets were collected from the well-developed spikelets in the middle of the spike. The entire endosperm was carefully removed from the grains (**Figure 1**). After freeze-drying for 48 h, each sample was weighed (to within 0.1 mg) and ground into a fine powder.

### 2.3 Content of total nitrogen

The total nitrogen contents of the caryopsis and endosperm samples during grain filling were calculated using a Kjeldahl™ 8400 nitrogen determinator (FOSS, Denmark). Briefly, each sample, weighing 100mg, was digested at a temperature of 420°C for 2 hours along with 3.6g of K_2_SO_4_, 0.4g of CuSO_4_·5H_2_O, and 8.0mL of H_2_SO_4_. After digestion, the cooled samples were analyzed for total nitrogen content.

### 2.4 Free amino acids

Samples of freeze-dried caryopsis and endosperm powder were steeped in 0.1 M HCl for 24 h before filtering through a 0.45-m membrane. FAAs were analyzed using an A300 amino acid analyzer (Membera Pure GmbH, Germany) after the filtrate was combined with an equivalent amount of 4% sulfosalicylic acid and filtered. Seventeen amino acids were identified, evaluated, and expressed in units of dry weight.

### 2.5 Contents of protein fractions

SE-HPLC was performed to assess the glutenin, gliadin, albumin, and globulin contents according to a method in the literature with slight modifications (Morel et al., 2000; Wang et al., 2017). Caryopsis and endosperm samples were suspended in 0.5% SDS, 0.1M phosphate buffer (pH 6.9) to extract the SDS-soluble proteins. The remaining proteins were resuspended in the same buffer after sonication for 30 s to extract the SDS-insoluble proteins. The SE-HPLC analysis was performed for 20 min at a 0.7 mL/min flow rate and 210 nm absorbance on TSK G4000-SWXL (7.8 × 300 mm, Tosoh Biosep) columns in a 0.02 M sodium phosphate buffer (pH 6.9). Three major peaks, F1–F3, were obtained from the SDS-soluble protein fraction. The SDS-insoluble protein fraction yielded F1 –F3*. The F1 and F1* peaks represent polymeric glutenins, F2 and F2* represent monomeric gliadins, and F3 and F3* represent albumins and globulins.

### 2.6 Statistical analysis

Differences were examined using SPSS (version 26.0; IBM, USA) for analysis of variance(ANOVA). The ANOVA mean comparisons were in terms of the least significant difference(LSD), and results with P < 0.05 were considered to show a statistically significant difference. Figures were drawn using OriginPro 2023 software (OriginLab, USA). The data in figures and tables were the average of three replicates.

## 3 Results

### 3.1 Accumulation of dry mass and total nitrogen in caryopsis and endosperm during grain development

**Figure 2:**
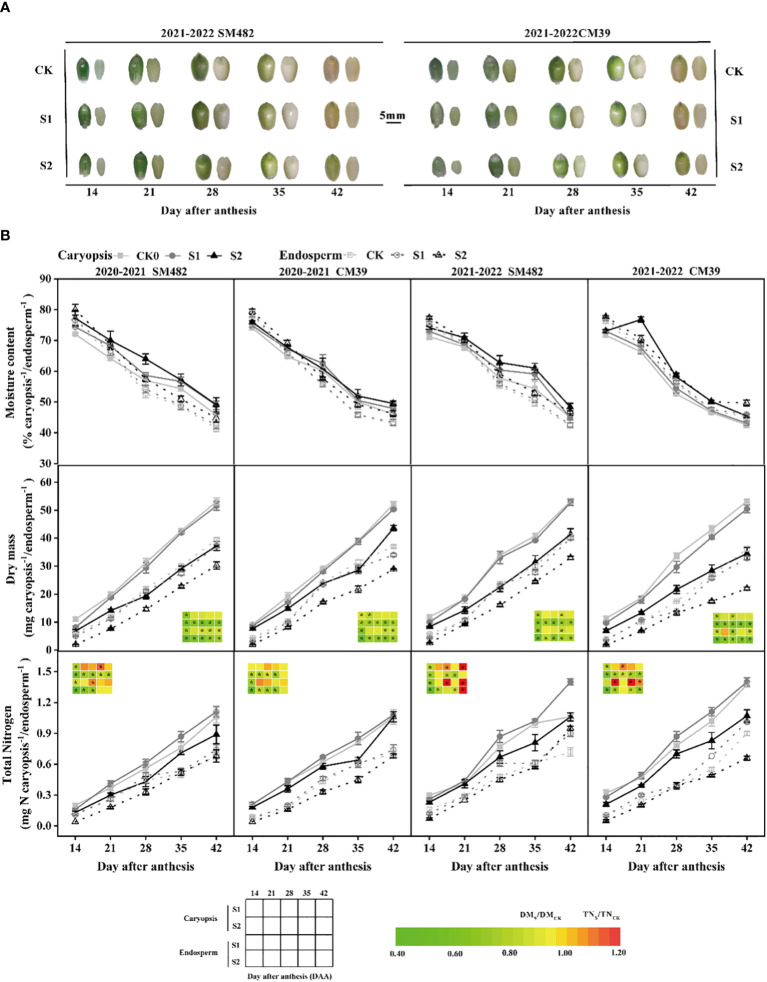
Changes in morphogenesis **(A)**, moisture content, dry mass, and total nitrogen accumulation **(B)** after anthesis in caryopsis and endosperm development. B The dry mass and total N difference in caryopsis and endosperm are converted to a color, calculated by shade stress divided by control treatment, and * in the figure indicate significant differences at p<0.05.

Shade stress delayed the growth of the caryopsis and endosperm (**Figure 2A**). At 14 DAA, the shade treatment showed much less development than the control. S1 showed a trend similar to that of CK after 21 DAA, whereas S2 still demonstrated a notable delay. The moisture levels in the caryopsis and endosperm was elevated by shade stress during grain filling (**Figure 2B**). For instance, the moisture content of S1 was similar to that of CK in the endosperm but greater than that of CK in the caryopsis, and the moisture content of S2 was higher than that of CK and S1 in the caryopsis and endosperm. The dry mass of S1 and S2 in the caryopsis was approximately 0.73–1.00 and 0.61–0.83 times that of CK, respectively, and that of S1 and S2 in the endosperm was approximately 0.76–1.01 and 0.33–0.88 times that of CK (**Figure 2B**). Notably, the dry matter weights both in caryopsis and endosperm were significantly lower than that of CK at 14-42DAA. The variance in the total nitrogen content differed from that in the dry mass, and the contrast between the shaded and control treatments was minor. The content of total nitrogen was significantly decreased by shade stress at 14 DAA. However, S1 exhibited higher total nitrogen content than CK from 21 to 42 DAA, but S2 continued to show a significantly downward trend(except SM482’s endosperm in 2021-2022). The amount of total nitrogen of S1 and S2 present in the caryopsis was approximately 0.80–1.32 and 0.65–1.00 times that of CK, respectively, and that in the endosperm of S1 and S2 was approximately 0.75–1.27 and 0.33–1.31 times that of CK. The results indicate that the S1 treatment positively influenced the accumulation of total nitrogen in the caryopsis and endosperm while slightly reduced the dry mass during grain filling; conversely, the S2 treatment had a detrimental effect on dry mass and total nitrogen buildup.

### 3.2 Free amino acid content in wheat caryopsis and endosperm

**Figure 3:**
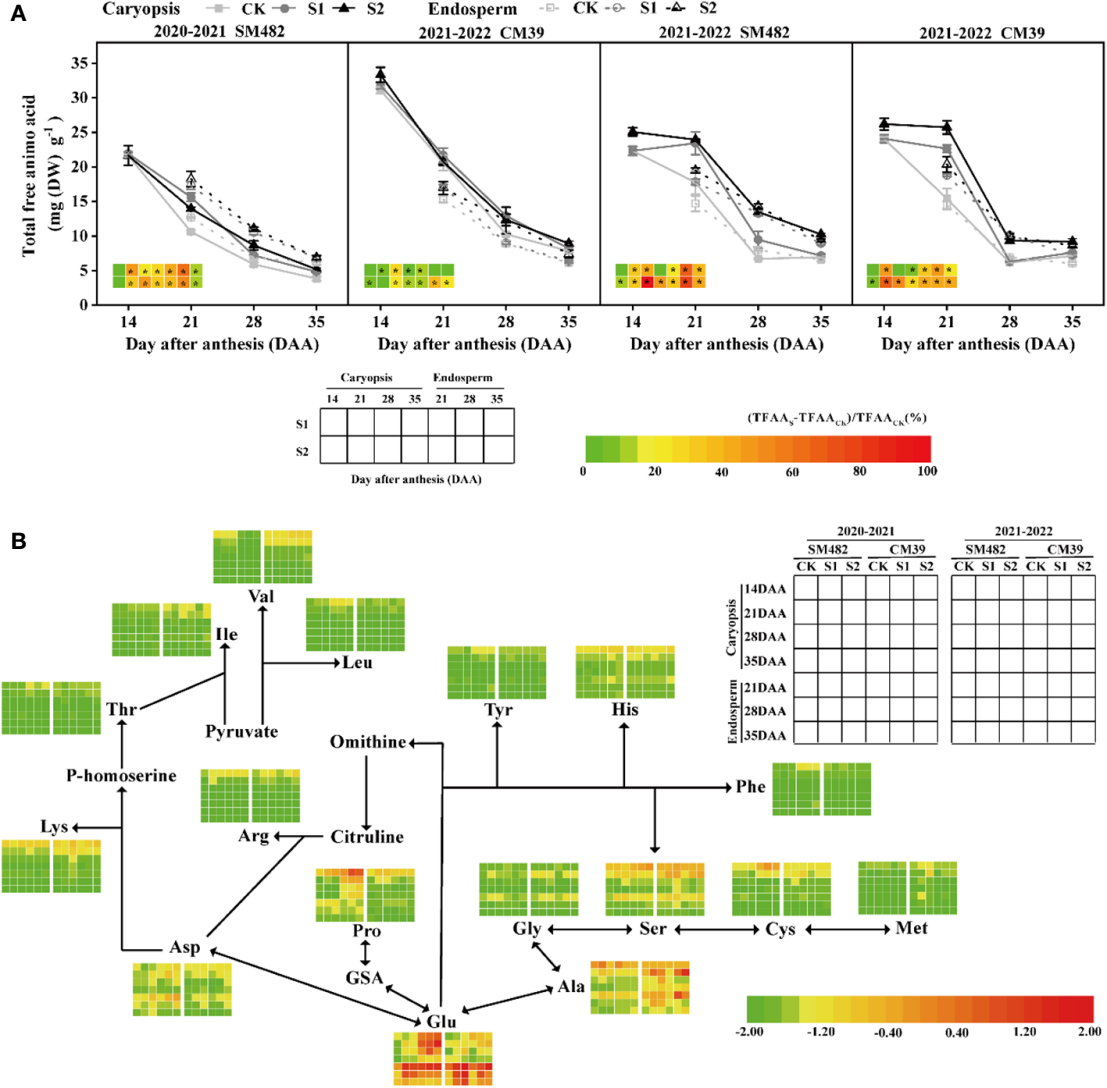
Changes in total free amino acids (TFAA) **(A)** and individual compositions **(B)** in wheat caryopsis and endosperm after anthesis under shade stress. Caryopsis free amino acid (FAA) analysis is performed 14–35 d after anthesis (DAA), and endosperm FAA analysis is performed 21–35 DAA. The TFAA difference in caryopsis and endosperm is converted to a color, calculated by the difference between shade stress (TFAA_S_) and control treatment (TFAA_CK_) divided by TFAA_CK_, and * in the figure indicate significant differences at p<0.05. B The heatmap of individual FAA during grain development is shown as normalized data rather than its actual content.

The wheat caryopsis and endosperm during the grain-filling stage are shown in **Figure 3**, along with the individual components and total quantities of FAAs. The concentration of total free amino acids (TFAA) in the wheat caryopsis and endosperm was significantly increased by shade stress during the filling stage (**Figure 3A**), and the increase in S2 was more pronounced than that in S1. As filling was maintained, the difference in TFAA between S1 and the CK in the caryopsis diminished, but the difference remained substantial in the endosperm. For the period 2021–2022, the TFAA content of the endosperm and caryopsis was higher in S1 by 20.6–66.5% and by 0.2–46.9%, respectively, than that of CK and was higher in S2 by 32.5–79.3% and by 12.3–100.7%. S2 and S1 exhibited comparable levels of TFAA in the endosperm. However, the two growing seasons showed different results in caryopsis, reflecting that the variation between S1 and S2 was less pronounced in 2020–2021 and the levels of TFAA were notably lower in S1 in 2021–2022.

A heatmap of individual FAAs in the caryopsis and endosperm during grain development is shown in **Figure 3B**. During grain filling, Glu, Ala, Ser, Gly, Asp, and Pro were the main FAAs in the caryopsis and endosperms, and Lys, His, Cys, and Arg were relatively high only in the caryopsis. Individual FAA levels in caryopsis consistently decreased from 14 DAA, and only Glu and Ala had high levels at 35 DAA. From 21 to 35 DAA, the individual amino acid content in the endosperm similarly changed with that in the caryopsis, and Glu was more abundant in the endosperm than in the caryopsis. During caryopsis and endosperm development, the shade stress groups had higher concentrations of Glu, Ser, Ala, and Asp than the control group did in both growing seasons. Besides, an increase in Gly occurred at 21 DAA after shade stress, and the increase in Pro occurred in 2020–2021. The amounts of each FAA in S1 and S2 were similar, except for Asp, which was typically greater in S2 than in S1, and Ile, which was higher in S2 than S1 in caryopsis at 14-21 DAA.

### 3.3 Variation in SDS-soluble and SDS-insoluble protein content during grain development

**Figure 4:**
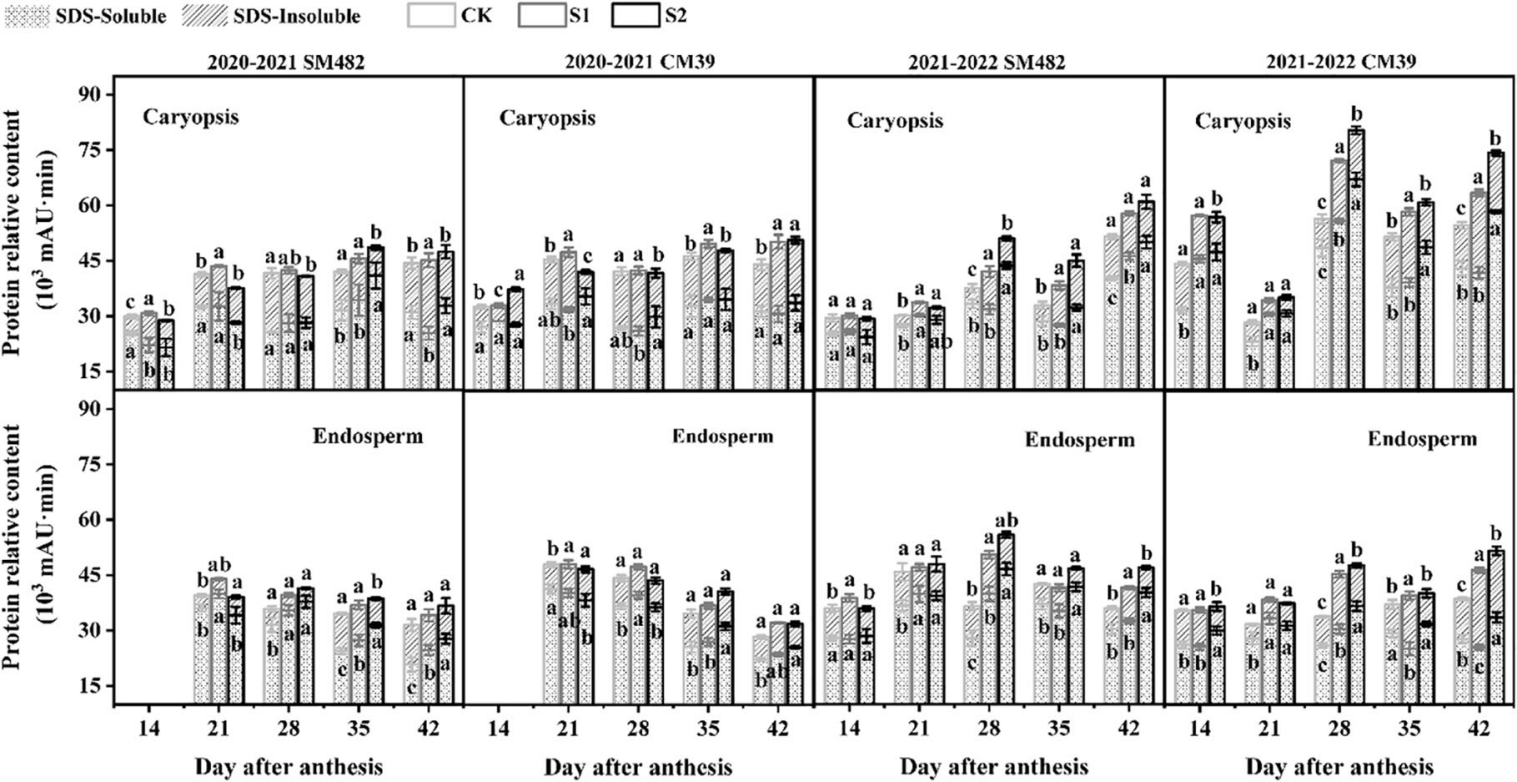
Changes in SDS-soluble and SDS-insoluble protein extracts under shade stress in wheat caryopsis and endosperm during grain filling. SDS-soluble (SDS-S) and SDS-insoluble (SDS-I) protein extracts were analyzed 14–42 DAA in caryopsis and endosperm (except in endosperm at 14 DAA in 2020–2021). The difference in SDS-S and SDS-I protein content is marked by the letters within and above the column, respectively. The different letters show a significant difference at P < 0.05.

During grain development, caryopsis and endosperm protein samples were extracted stepwise using SDS and analyzed by SE-HPLC. **Figure 4** shows the changes in the two protein fractions in response to shade-induced stress in the caryopsis and endosperm during the grain-filling period. Regarding the observed elevation in total protein levels in shade stress treatments compared with that in the control group (CK), that of S1 was ascribed to an increase in SDS-insoluble (SDS-I) protein extracts, and that of S2 was the increase in SDS-soluble (SDS-I) protein extracts. In caryopsis, the SDS-S protein fraction was higher in S2 than in the CK treatment, especially in 2021–2022, and the SDS-I protein fraction was the highest in S1, which was considerably more significant than in the CK treatment. Alterations in the protein components in the endosperm were similar to those in the caryopsis, and there were apparent distinctions among S1, S2, and the CK in 2021–2022. The SDS-S component of S1 showed a decreasing trend with S2, but the SDS-I component of S1 showed an increasing trend with S2, with substantial variances between S1 and S2 in the caryopsis and endosperm at 28-42 DAA in 2021–2022.

### 3.4 Variation in protein composition during grain development

**Figure 5:**
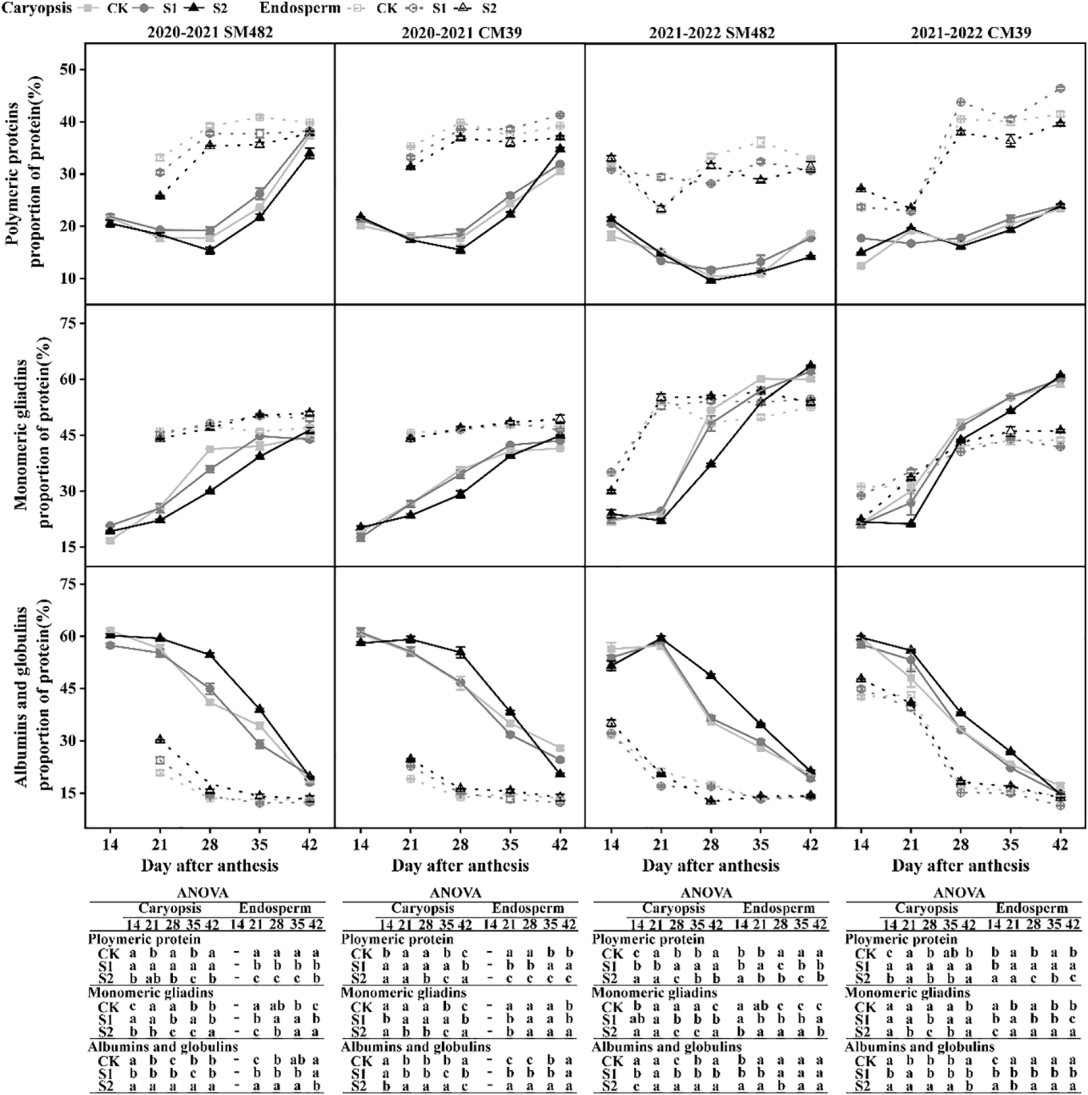
Changes in polymeric and monomeric proteins proportion under shade stress of wheat caryopsis and endosperm during grain development. The protein proportion was analyzed 14–42 DAA in caryopsis and endosperm (except in endosperm at 14 DAA in 2020–2021, and the different letters show a significant difference at P < 0.05.

Alterations in the composition of proteins in the caryopsis and endosperm after shade stress are depicted in **Figure 5** for the filling stage. The polymer and monomer protein contents of the caryopsis and endosperm during the filling stage were consistent in the two growth seasons. There was a difference in the polymeric gluteins and monomeric gliadins in the endosperm between the two cultivars.

#### 3.4.1 Polymeric proteins

The polymeric protein of S1 exhibited an increase than that of CK within the caryopsis, and S2 showed a decrease. However, the polymer protein of S2 was significantly higher than that of CK at 42 DAA in CM39. At 28–42 DAA, the concentration of the polymeric protein in the CK was significantly higher than that of S1 and S2 in the endosperm of SM482, and S1 had a higher concentration than that of the CK and S2 in the endosperm of CM39. In the SM482 variety, the disparity between the shade treatment and the control became progressively minor at 35-42 DAA.

#### 3.4.2 Monomeric gliadins

The monomeric protein component of S2 in caryopsis was significantly less than those of the CK, and those of S1 were slightly less than those of the CK from 21 to 35 DAA. However, the monomeric protein content was increased by shade stress at 42 DAA. Additionally, there was a greater increase in monomeric gliadins in S2 than in the CK and S1. From 28 to 42 DAA, the monomeric protein in endosperm of S1 in SM482 was significantly lower than that in CK, but that in CM39 showed an increase than CK.

#### 3.4.3 Albumins and globulins

The albumins and globulins levels of S2 were higher than those of the CK and S1 in the endosperm, especially in 2020-2021. In caryopsis, the levels of albumins and globulins in S1 were similar to those in the CK, and the content of albumins and globulins of S2 from 21 to 35 DAA was considerably higher than that of the CK and S1. The albumin and globulin levels in S2 were lower than those in the CK at 42 DAA.

The results indicate that the S1 treatment positively influenced the accumulation of polymeric protein in the caryopsis during grain filling and in the endosperm at the late filling stage; conversely, the S2 treatment had a detrimental effect on polymeric protein buildup at early and mid filling stage.

### 3.5 Variation in SDS-unextractable polymeric protein during grain filling

**Figure 6:**
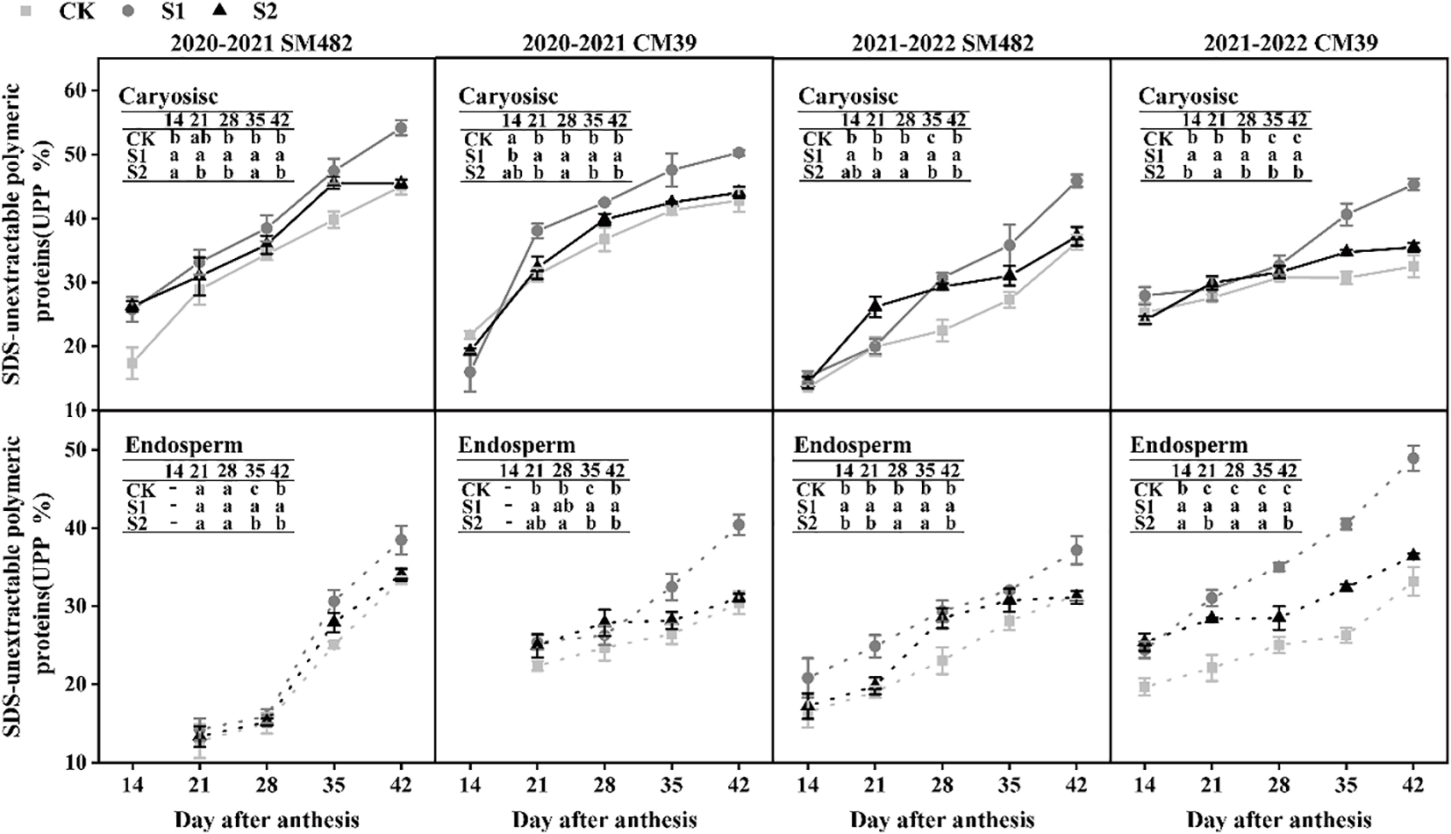
Changes in SDS-unextractable polymeric proteins (UPP) under shade stress of wheat caryopsis and endosperm during grain development. UPP were analyzed 14–42 d after anthesis in caryopsis and endosperm (except in endosperm at 14 DAA in 2020–2021). UPP is calculated by dividing the SDS-insoluble polymer protein by the polymer protein, and the different letters show a significant difference at P < 0.05.

**Figure 6** illustrates the changes in SDS-unextractable polymeric protein (UPP) in the caryopsis and endosperm during the filling stage under shade stress. The UPP content was significantly increased by shade stress during grain filling. The UPP of S1 in caryopsis was significantly higher than that of the CK and S2 from 21 to 42 DAA. Additionally, S2 showed more UPP content than CK. The rate of increase in UPP in S2 was lower than that in the CK from 35 to 42 DAA, but S1 rapidly increased in UPP. The UPP content in the endosperm was identical to that in the caryopsis. The shade treatments exhibited greater values than those of the CK from 14 to 42 DAA. The UPP of S2 increased slowly but remained higher than that of CK from 28 to 42 DAA.

## 4 Discussion

Grain filling is crucial for growth and extremely responsive to environmental changes (Teng et al., 2023). Wheat showed a trend of decreasing grain weight when the amount of assimilates available was constrained by abiotic factors (Alvarez Prado et al., 2023). Our result that the dry mass of caryopsis and endosperm was reduced by shade stress during grain filling supports the results of Artru et al. (2017), Yang et al. (2023b) and Li et al. (2012). The reduction of grain weight under shading from the joint to mature stage treatment (S2) was more than that of shading from the joint to anthesis stage treatment (S1) and was due to its constant shading environment in which assimilates produced by photosynthesis decreased during grain filling (Li et al., 2010). Another study has demonstrated that shade can reduce the effect on grain weight by modifying the allocation of nutrients from the plant’s reserves to the grains (Maydup et al., 2020), and that has been found to enhance the total nitrogen content(%) in grains (Jia et al., 2021). Additionally, consistent with those aforementioned results: the S1 treatment increased the accumulation of total nitrogen content in caryopsis and endosperm, and the S2 treatment had a detrimental influence on total nitrogen buildup due to the dry mass decreased sharply (**Figure 2**).

FAAs are required for protein biosynthesis and have essential functions in other metabolic pathways and signal transduction processes; their content and composition may change dynamically and substantially in response to environmental factors or developmental stages (Hildebrandt et al., 2015). During grain filling, the TFAA content steadily decreased (**Figure 3A**), as in Zhong et al. (2018). Shade stress increased the TFAA in caryopsis and endosperm during grain development more than it did in the CK. The TFAA of S2 was higher than that of S1 because the decrease in dry mass of S2 was more pronounced than that of S1 (**Figure 3A**), and thus its FAA was less diluted by starch and gluten proteins (Shi et al., 2019). As filling was maintained, the difference between S1 and the CK in caryopsis diminished, but the difference remained substantial in the endosperm. The starchy endosperm uses amino acids for gliadin and glutenin synthesis, and increasing amino acid delivery and availability increases the protein content in the endosperm (Wan et al., 2021). In our study, during caryopsis and endosperm development, shade stress groups had higher concentrations of Glu, Ser, Ala, and Asp than the control group did (**Figuer 3B**), which are the major components for storage protein synthesis (Zhong et al., 2020). Aspartic and glutamic acids are important substrates for nitrogen (N) supply (Zhang et al., 2021), Glu was similar in S1 and S2, and S2 contained more Asp than S1 did. The increase in these FAAs may contribute to wheat protein content (Zhen et al., 2016).

The quantity and proportion of proteins in developing grains can also be affected by environmental conditions such as precipitation or global radiation (Wroblewitz et al., 2014). When abiotic (cloudy periods, global dimming) adversities occur, protein accumulation in grains can be partially regulated by the availability of assimilates post-flowering (Arata et al., 2023). Artru et al. (2017) and Yang et al. (2023a) have found that shade stress increases protein in mature grains and flour. In our results, shade stress increased the total protein content during caryopsis and endosperm growth (**Figure 4**). There is a distinct correlation between grain moisture loss and protein polymerization, and grains with a higher moisture content may contain a lower proportion of SDS-insoluble protein (Johansson et al., 2008). During grain filling, S1 had a moisture content similar to that of the CK, and S2 had a moisture content higher than that of the CK (**Figure 2B**). A possible reason for the increase in total protein is that the S2 protein was ascribed to the increase in the SDS-soluble protein extracts, and the S1 protein was ascribed to the increase in the SDS-insoluble protein extracts.

On the basis of protein aggregating properties, wheat proteins may be classified as polymeric (glutenins) and monomeric (gliadins, albumins, and globulins) (Gupta et al., 1996). Glutenins and gliadins are the primary components of wheat’s storage protein, which affect dough viscoelasticity and baking quality (PanozzoA et al., 2001). In wheat grains, glutenins and gliadins are accumulated and co-located in the endosperm (Tosi et al., 2011). Glutenins and gliadins accumulated rapidly before 35 DAA and slowed down after 42 DAA during grain filling (Shewry et al., 2009), and the proportion of albumins and globulins decreased continuously (Koga et al., 2017). Another study found that the gliadins and glutenins contents of flour increased after different shading periods (Yang et al., 2023a). Our results further reveal that S1 promoted the formation of polymer protein in the caryopsis and endosperm, and S2 increased the proportion of monomeric gliadins at 42 DAA (**Figure 5**). S2 exhibited lower levels of gliadin and glutenin and greater levels of albumin and globulin before 42 DAA than CK and S1 did. However, the difference in glutenin content between S2 and the CK and S1 was reduced at 42 DAA, and S2 exhibited a higher level of gliadin and lower levels of albumin and globulin than CK and S1 did (**Figure 5**). The pattern of protein synthesis is characterized by the accumulation of one protein class and the decrease in another protein class (PanozzoA et al., 2001) during grain filling. Albumin and globulin may be precursors for the synthesis of glutenins and gliadins (Stone and Nicolas, 1996; Ferreira et al., 2012). This observation could be partly explained by the delayed caryopsis and endosperm development of S2 (**Figure 2**), which causes the conversion of albumin and globulin into monomeric and polymeric forms to be delayed (**Figure 5**).

SDS-unextractable polymeric protein as a percentage of UPP was used to represent the glutenin polymer size distribution, which was positively correlated with the rheological properties (Gupta et al., 1993). Variations in UPP have been applied to reflect the process of quality formation during grain filling (Koga et al., 2017; Gao et al., 2018). In our study, shade stress increased the UPP of the caryopsis and endosperms during grain filling (**Figure 6**). Grain desiccation could enhance the fraction of SDS-insoluble polymers due to an increase in the proportion of HMW-GS in glutenins (Carceller and Aussenac, 2001). The SDS-insoluble gel of glutenin polymers is called glutenin macropolymer (GMP) (Don et al., 2003). Li et al. (2012) reported that shading from the joint to the mature stage influences HMW and GMP accumulation during grain filling, which may support our results. The accumulation of polymeric proteins during grain development is highly influenced by the growing circumstances (Aussenac et al., 2020) The size of the glutenin polymer decreased during grain desiccation, possibly due to the increase in moisture content disturbing the polymer assembly (Koga et al., 2020). The polymerization of polymers was related to the continuous dehydration after physiological maturity (Ferrise et al., 2015) and the rapid water loss during grain desiccation (Carceller and Aussenac, 2001). After the transition of grain color from green to yellow during grain filling, the size of polymeric proteins increased (Koga et al., 2017). Similar results confirmed that the increase in UPP in S2 was slower than in the CK and S1 in our results, partly explained by the slightly green grain at 42 DAA and the high moisture content of caryopsis from 35 to 42 DAA for S2 (**Figure 6**). In the literature, the accumulation of UPP increased flour processing quality (Li et al., 2019; Olckers et al., 2022) and was a good predictor of bread-making quality even under abiotic stress (Olckers et al., 2022).

## 5 Conclusions

**Figure 7:**
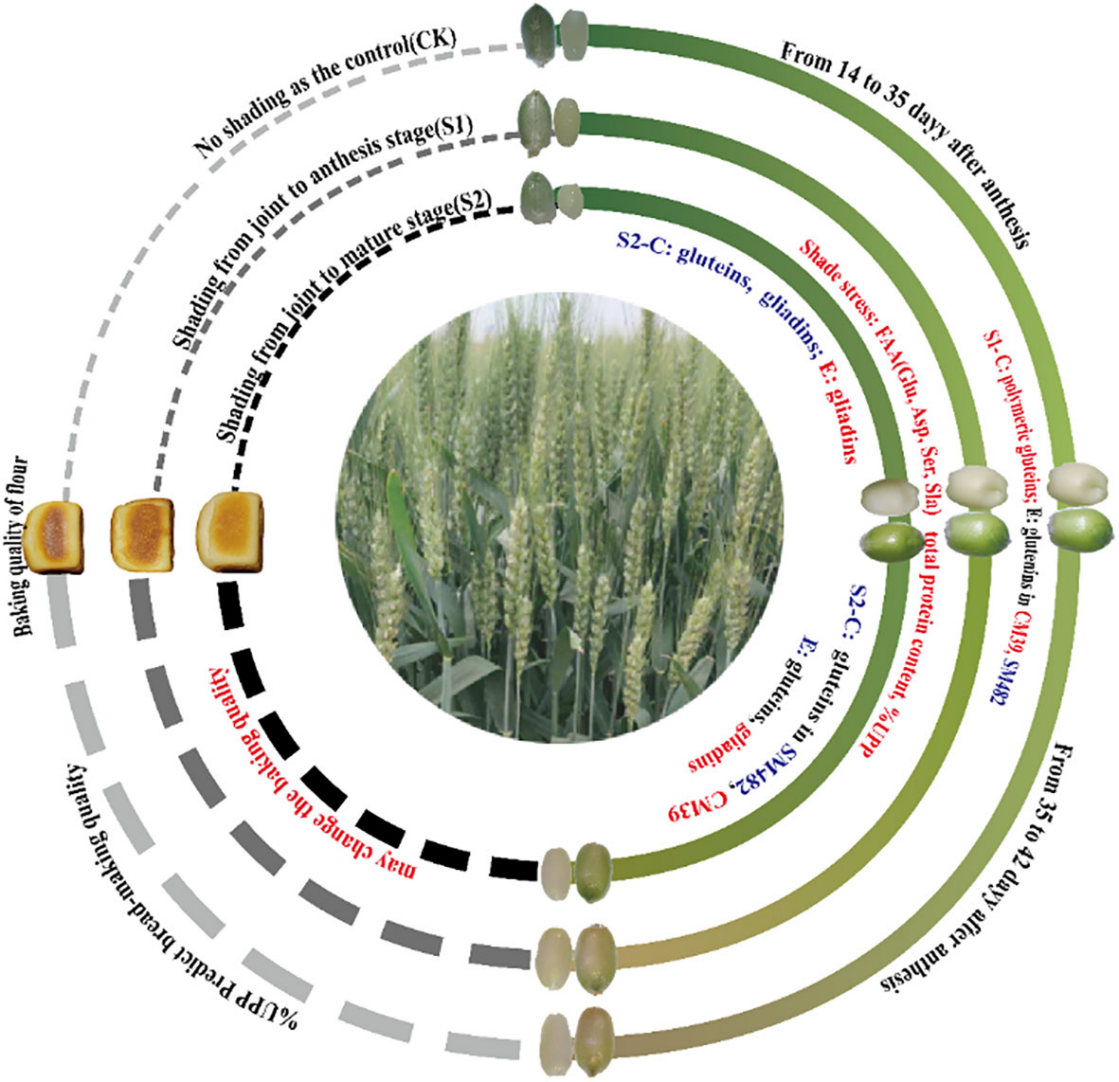
Changes in protein accumulation and polymerization of wheat after shading. C, caryopsis; E, endosperm; FAA, free amino acid; UPP, Unextractable polymeric protein. Dark blue indicates a decreasing trend between shade stress and the control; red indicates an increasing trend between shade stress and the control; black indicates a similar trend between shade stress and the control.

Analysis of the changes in the free amino acid and protein accumulation and polymerization in caryopsis and endosperm is essential for understanding the mechanism underlying the establishment of wheat quality formation in response to shade stress. This study showed that shade stress influenced protein accumulation and polymerization (**Figure 7**). Shade stress increased the FAA supply levels and improved Glu, Asp, Ala, and Ser. The increase in the relative protein content of S2 was ascribed to the increase in SDS-S protein extracts, and that of S1 was ascribed to the increase in SDS-I protein extracts. Further analysis revealed that S1 promoted the formation of polymer proteins in the caryopsis and endosperm. In contrast, S2 delayed polymeric glutein synthesis, and monomeric gliadins were promoted in the endosperm. Shade stress increased the accumulation of UPP in the caryopsis and endosperm during grain filling, which represented an increase in the degree of glutein polymerization. These findings provide novel insights into wheat quality formation under shade stress by protein accumulation and polymerization of the caryopsis and endosperm.

## Data availability statement

The original contributions presented in the study are included in the article/supplementary material. Further inquiries can be directed to the corresponding authors.

## Author contributions

HM: Data curation, Formal analysis, Investigation, Writing – original draft, Writing – review & editing. YY: Investigation, Methodology, Writing – original draft. DW: Investigation, Methodology, Writing – original draft. GX: Data curation, Methodology, Writing – review & editing. TL: Data curation, Writing – review & editing. XH: Investigation, Writing – original draft. HY: Methodology, Writing – original draft. TZ: Conceptualization, Writing – review & editing. GF: Funding acquisition, Supervision, Writing – review & editing.

## References

[B1] Alvarez Prado S. Giménez V. D. Ciancio N. Alzueta I. Serrago R. A. Miralles D. J. (2023). Grain growth and development in wheat (*Triticum aestivum* L.) and barley (*Hordeum vulgare* L.): Coordination between water content and source/sink ratio. Field Crop Res. 302, 109100. doi: 10.1016/j.fcr.2023.109100

[B2] Arata A. F. Lázaro L. Tranquilli G. E. Arrigoni A. C. Martínez M. Rondanini D. P. (2023). How does post-flowering source/sink manipulation affect grain weight and commercial quality in argentinean bread wheat genotypes with different baking aptitude? Field Crop Res. 301, 109030. doi: 10.1016/j.fcr.2023.109030

[B3] Artru S. Garré S. Dupraz C. Hiel M. Blitz-Frayret C. Lassois L. (2017). Impact of spatio-temporal shade dynamics on wheat growth and yield, perspectives for temperate agroforestry. Eur. J. Agron. 82, 60–70. doi: 10.1016/j.eja.2016.10.004

[B4] Aussenac T. Rhazi L. Branlard G. (2020). Molecular weight distribution of polymeric proteins in wheat grains: the rheologically active polymers. Foods 9, 1675. doi: 10.3390/foods9111675 33207650 PMC7697747

[B5] Bechtel D. B. Abecassis J. Shewry P. R. Evers A. D. (2009). “Development, structure, and mechanical properties of the wheat grain,” in Wheat (Fourth Edition) (St. Paul, Minnesota 55121, U.S.A: AACC International), 51–95.

[B6] Carceller J. L. Aussenac T. (2001). SDS-insoluble glutenin polymer formation in developing grains of hexaploid wheat: the role of the ratio of high to low molecular weight glutenin subunits and drying rate during ripening. Aust. J. Plant Physiol. 28, 193–201. doi: 10.1071/PP00002

[B7] Daniel C. Triboi E. (2002). Changes in wheat protein aggregation during grain development: effects of temperatures and water stress. Eur. J. Agron. 16, 1–12. doi: 10.1016/S1161-0301(01)00114-9

[B8] Don C. Lichtendonk W. Plijter J. J. Hamer R. J. (2003). Glutenin macropolymer: a gel formed by glutenin particles. J. Cereal Sci. 37, 1–7. doi: 10.1006/jcrs.2002.0481

[B9] Dowell F. E. Maghirang E. B. Pierce R. O. Lookhart G. L. Bean S. R. Xie F.. (2008). Relationship of bread quality to kernel, flour, and dough properties. Cereal Chem. 85, 82–91. doi: 10.1094/CCHEM-85-1-0082

[B10] Ferreira M. S. L. Samson M. Bonicel J. Morel M. (2012). Relationship between endosperm cells redox homeostasis and glutenin polymers assembly in developing durum wheat grain. Plant Physiol. Biochem. 61, 36–45. doi: 10.1016/j.plaphy.2012.08.015 23031846

[B11] Ferrise R. Bindi M. Martre P. (2015). Grain filling duration and glutenin polymerization under variable nitrogen supply and environmental conditions for durum wheat. Field Crop Res. 171, 23–31. doi: 10.1016/j.fcr.2014.10.016

[B12] Gao X. Liu T. Ding M. Wang J. Li C. Wang Z.. (2018). Effects of hmw-gs ax1 or dx2 absence on the glutenin polymerization and gluten micro structure of wheat (*Triticum aestivum* L.). Food Chem. 240, 626–633. doi: 10.1016/j.foodchem.2017.07.165 28946321

[B13] Gupta R. B. Khan K. Macritchie F. (1993). Biochemical basis of flour properties in bread wheats. I. Effects of variation in the quantity and size distribution of polymeric protein. J. Cereal Sci. 18, 23–41. doi: 10.1006/jcrs.1993.1031

[B14] Gupta R. B. Stefania M. Domenico L. Bariana H. S. Finlay M. R. (1996). Accumulation of protein subunits and their polymers in developing grains of hexaploid wheats. J. Exp. Bot. 47, 1377–1385. doi: 10.1093/jxb/47.9.1377

[B15] Han W. Lin X. Wang. D. (2023). Uncovering the primary drivers of regional variability in the impact of climate change on wheat yields in China. J. Clean Prod. 421, 138479. doi: 10.1016/j.jclepro.2023.138479

[B16] Hayta M. Schofield J. D. (2004). Heat and additive induced biochemical transitions in gluten from good and poor breadmaking quality wheats. J. Cereal Sci. 40, 245–256. doi: 10.1016/j.jcs.2004.06.006

[B17] Hildebrandt T. M. Nesi A. N. Araujo W. L. Braun H. (2015). Amino acid catabolism in plants. Mol. Plant 8, 1563–1579. doi: 10.1016/j.molp.2015.09.005 26384576

[B18] Jia J. Xu M. Bei S. Zhang H. Xiao L. Gao Y.. (2021). Impact of reduced light intensity on wheat yield and quality: implications for agroforestry systems. Agrofor. Syst. 95, 1689–1701. doi: 10.1007/s10457-021-00668-w

[B19] Johansson E. Prieto-Linde M. L. Gissen C. (2008). Influences of weather, cultivar and fertiliser rate on grain protein polymer accumulation in field-grown winter wheat, and relations to grain water content and falling number. J. Sci. Food Agric. 88, 2011–2018. doi: 10.1002/jsfa.3312

[B20] Koga S. Aamot H. U. Uhlen A. K. Seehusen T. Veiseth-Kent E. Hofgaard I. S.. (2020). Environmental factors associated with glutenin polymer assembly during grain maturation. J. Cereal Sci. 91, 102865. doi: 10.1016/j.jcs.2019.102865

[B21] Koga S. Böcker U. Wieser H. Koehler P. Uhlen A. K. Moldestad A. (2017). Polymerisation of gluten proteins in developing wheat grain as affected by desiccation. J. Cereal Sci. 73, 122–129. doi: 10.1016/j.jcs.2016.12.003

[B22] Li H. Jiang D. Wollenweber B. Dai T. Cao W. (2010). Effects of shading on morphology, physiology and grain yield of winter wheat. Eur. J. Agron. 33, 267–275. doi: 10.1016/j.eja.2010.07.002

[B23] Li X. Cai J. Li H. Bo Y. Liu F. Jiang D.. (2012). Effect of shading from jointing to maturity on high molecular weight glutenin subunit accumulation and glutenin macropolymer concentration in grain of winter wheat. J. Agron. Crop Sci. 198, 68–79. doi: 10.1111/j.1439-037X.2011.00484.x

[B24] Li S. Wang J. Ding M. Min D. Wang Z. Gao X. (2019). The influence of night warming treatment on the micro-structure of gluten in two wheat cultivars. Food Res. Int. 116, 329–335. doi: 10.1016/j.foodres.2018.08.043 30716953

[B25] Li Y. Tao F. (2022). Interactions of genotype, environment and management on wheat traits and grain yield variations in different climate zones across China. Agric. Syst. 203, 103521. doi: 10.1016/j.agsy.2022.103521

[B26] Liu J. Wu M. Liu C. (2022). Cereal endosperms: development and storage product accumulation. Annu. Rev. Plant Biol. 73, 255–291. doi: 10.1146/annurev-arplant-070221-024405 35226815

[B27] Ma H. Yang Y. Zhao J. Huang X. Yang H. Zheng T.. (2024). Relationship between the baking quality of wheat (*Triticum aestivum* L.) And the protein composition and structure after shading. Food Chem. 441, 138392. doi: 10.1016/j.foodchem.2024.138392 38211475

[B28] Maydup M. L. Antonietta M. Rouillet N. Cano M. G. Guiamet J. J. Tambussi E. A. (2020). Methodological aspects and impact on grain weight of source reduction through shade meshes during grain filling of bread wheat. Crop Pasture Sci. 71, 739–751. doi: 10.1071/CP18565

[B29] Morel M. H. Dehlon P. Autran J. C. Leygue J. P. Helgouac’H B. (2000). Effects of temperature, sonication time, and power settings on size distribution and extractability of total wheat flour proteins as determined by size-exclusion high-performance liquid chromatography. Cereal Chem. 5, 685–691. doi: 10.1094/CCHEM.2000.77.5.685

[B30] Nuttall J. G. O’Leary G. J. Panozzo J. F. Walker C. K. Barlow K. M. Fitzgerald G. J. (2017). Models of grain quality in wheat—a review. Field Crop Res. 202, 136–145. doi: 10.1016/j.fcr.2015.12.011

[B31] Olckers S. Osthoff G. Guzmán C. Wentzel B. van Biljon A. Labuschagne M. (2022). Drought and heat stress effects on gluten protein composition and its relation to bread-making quality in wheat. J. Cereal Sci. 108, 103562. doi: 10.1016/j.jcs.2022.103562

[B32] PanozzoA J. F. EaglesA H. A. Wootton M. (2001). Changes in protein composition during grain development in wheat. Crop Pasture Sci. 52, 485–493. doi: 10.1071/AR00101

[B33] Pramanick B. Kumar M. Naik B. M. Kumar M. Singh S. K. Maitra S.. (2022). Long-term conservation tillage and precision nutrient management in maize-wheat cropping system: effect on soil properties, crop production, and economics. Agronomy-Basel 12, 2766. doi: 10.3390/agronomy12112766

[B34] Qiao X. Sai L. Chen X. Xue L. Lei J. (2019). Impact of fruit-tree shade intensity on the growth, yield, and quality of intercropped wheat. PloS One 14, e0203238. doi: 10.1371/journal.pone.0203238 30939172 PMC6445427

[B35] Qin W. Wang L. Gueymard C. A. Bilal M. Lin A. Wei J.. (2020). Constructing a gridded direct normal irradiance dataset in China during 1981-2014. Renew. Sust. Energ. Rev. 131. doi: 10.1016/j.rser.2020.110004

[B36] Shewry P. R. Morell M. (2001). Manipulating cereal endosperm structure, development and composition to improve end-use properties. Adv. Bot. Res. 34, 165–236. doi: 10.1016/S0065-2296(01)34009-0

[B37] Shewry P. R. Underwood C. Wan Y. Lovegrove A. Bhandari D. Toole G.. (2009). Storage product synthesis and accumulation in developing grains of wheat. J. Cereal Sci. 50, 106–112. doi: 10.1016/j.jcs.2009.03.009

[B38] Shi Z. Wang Y. Wan Y. Hassall K. Jiang D. Shewry P. R.. (2019). Gradients of gluten proteins and free amino acids along the longitudinal axis of the developing caryopsis of bread wheat. J. Agric. Food Chem. 67, 8706–8714. doi: 10.1021/acs.jafc.9b02728 31310118

[B39] Stone P. J. Nicolas M. E. (1996). Varietal differences in mature protein composition of wheat resulted from different rates of polymer accumulation during grain filling. Funct. Plant Biol. 23, 727–737. doi: 10.1071/pp9960727

[B40] Teng Z. Chen Y. Meng S. Duan M. Zhang J. Ye N. (2023). Environmental stimuli: a major challenge during grain filling in cereals. Int. J. Mol. Sci. 24, 2255. doi: 10.3390/ijms24032255 36768575 PMC9917212

[B41] Tosi P. Gritsch C. S. He J. Shewry P. R. (2011). Distribution of gluten proteins in bread wheat (*Triticum aestivum*) grain. Ann. Bot. 108, 23–35. doi: 10.1093/aob/mcr098 21693664 PMC3119610

[B42] Tosi P. He J. Lovegrove A. Gonzáles-Thuillier I. Penson S. Shewry P. R. (2018). Gradients in compositions in the starchy endosperm of wheat have implications for milling and processing. Trends Food Sci. Technol. 82, 1–7. doi: 10.1016/j.tifs.2018.09.027 30532347 PMC6267945

[B43] Wan Y. Wang Y. Shi Z. Rentsch D. Ward J. L. Hassall K.. (2021). Wheat amino acid transporters highly expressed in grain cells regulate amino acid accumulation in grain. PloS One 16, e0246763. doi: 10.1371/journal.pone.0246763 33606697 PMC7894817

[B44] Wang X. Appels R. Zhang X. Bekes F. Torok K. Tomoskozi S.. (2017). Protein-transitions in and out of the dough matrix in wheat flour mixing. Food Chem. 217, 542–551. doi: 10.1016/j.foodchem.2016.08.060 27664670

[B45] Wroblewitz S. Huether L. Manderscheid R. Weigel H. Waetzig H. Daenicke S. (2014). Effect of rising atmospheric carbon dioxide concentration on the protein composition of cereal grain. J. Agric. Food Chem. 62, 6616–6625. doi: 10.1021/jf501958a 24976461

[B46] Yang X. Asseng S. Wong M. T. F. Yu Q. Li J. Liu E. (2013). Quantifying the interactive impacts of global dimming and warming on wheat yield and water use in China. Agric. For. Meteorol. 182, 342–351. doi: 10.1016/j.agrformet.2013.07.006

[B47] Yang H. Li Y. Zhao J. Chen Z. Huang X. Fan G. (2023a). Regulating the composition and secondary structure of wheat protein through canopy shading to improve dough performance and nutritional index. Food Res. Int. 173, 113399. doi: 10.1016/j.foodres.2023.113399 37803737

[B48] Yang H. Zhao J. Ma H. Shi Z. Huang X. Fan G. (2023b). Shading affects the starch structure and digestibility of wheat by regulating the photosynthetic light response of flag leaves. Int. J. Biol. Macromol. 236, 123972. doi: 10.1016/j.ijbiomac.2023.123972 36906208

[B49] Zhang S. Ghatak A. Bazargani M. M. Bajaj P. Varshney R. K. Chaturvedi P.. (2021). Spatial distribution of proteins and metabolites in developing wheat grain and their differential regulatory response during the grain filling process. Plant J. 107, 669–687. doi: 10.1111/tpj.15410 34227164 PMC9291999

[B50] Zhen S. Dong K. Deng X. Zhou J. Xu X. Han C.. (2016). Dynamic metabolome profiling reveals significant metabolic changes during grain development of bread wheat (*Triticum aestivum L.*). J. Sci. Food Agric. 96, 3731–3740. doi: 10.1002/jsfa.7561 26676564

[B51] Zheng B. Jiang J. Wang L. Huang M. Zhou Q. Cai J.. (2022). Reducing nitrogen rate and increasing plant density accomplished high yields with satisfied grain quality of soft wheat via modifying the free amino acid supply and storage protein gene expression. J. Agric. Food Chem. 70, 2146–2159. doi: 10.1021/acs.jafc.1c07033 35142500

[B52] Zhong Y. Vidkjær N. H. Massange-Sanchez J. A. Laursen B. B. Gislum R. Borg S.. (2020). Changes in spatiotemporal protein and amino acid gradients in wheat caryopsis after n-topdressing. Plant Sci. 291, 110336. doi: 10.1016/j.plantsci.2019.110336 31928684

[B53] Zhong Y. Xu D. Hebelstrup K. H. Yang D. Cai J. Wang X.. (2018). Nitrogen topdressing timing modifies free amino acids profiles and storage protein gene expression in wheat grain. BMC Plant Biol. 18, 353. doi: 10.1186/s12870-018-1563-3 30545290 PMC6293556

